# Diabetic ketoacidosis and high mortality among patients with coronavirus disease 2019 in a Peruvian hospital

**DOI:** 10.1111/jdi.13704

**Published:** 2021-11-23

**Authors:** Eddy Lopez‐Huamanrayme, Claudia Cordova‐Huancas, Dioni Garate‐Chirinos, Frank Espinoza‐Morales, Francisco Pasquel

**Affiliations:** ^1^ Division of Endocrinology Hospital Nacional Alberto Sabogal Sologuren Callao Peru; ^2^ Centro de Tecnologías Aplicadas a la Diabetes Lima Peru; ^3^ Department of Medicine Emory University School of Medicine Atlanta USA

## DISCLOSURE

FEM is a medical scientific liaison for SANOFI Peru. FJP is supported in part by NIH under Award Numbers 1K23GM12822101A1, P30DK111024 and P30DK11102405S1, and has received research support from Dexcom and Merck, and consulting fees from Boehringer Ingelheim. The other authors declare no conflict of interest.

Approval of the research protocol: The study protocol was approved by the Ethics Committee of the *Instituto de Evaluación de Tecnologías en Salud e Investigación* (Lima, Peru).

Informed consent: N/A.

Approval date of registry and the registration no. of the study: N/A.

Animal studies: N/A.

Dear Editor,

Patients with diabetes and coronavirus disease 2019 (COVID‐19) are vulnerable to the worst outcomes in hospital. Among patients with diabetic ketoacidosis (DKA) treated with standard of care continuous insulin infusion, the mortality reported was up to 30%^1^, significantly higher compared with pre‐pandemic data.

There are limited data from developing countries, such as Peru, which has the highest mortality per million inhabitants in the world. Through a virtual platform, we extracted individual data from patients admitted to Hospital Nacional Alberto Sabogal Sologuren (Callao, Peru) between March and December 2020 with COVID‐19 and DKA and/or hyperglycemic hyperosmolar state, to obtain clinical information and outcomes.

A total of 31 patients were included, 61.3% were men, the median age was 56 years, 41.9% of patients showed new diabetes and we did not have any patients with type 1 diabetes, the rest of the patients had type 2 diabetes. More than 80% of patients were diagnosed with DKA, and no patient with isolated hyperglycemic hyperosmolar state was found. Other clinical and laboratory characteristics are shown in Table 1.

**Table 1:** Mortality and characteristics of patients with diabetic ketoacidosis hospitalized for coronavirus disease 2019

Variables		Mortality		
	*n* = 31	Yes (*n* = 23)	No (*n* = 8)	Unadjusted P*
	*n* (%)	*n* (%)	*n* (%)	
Male	19 (61.3)	14 (73.7)	5 (26.3)	0.62
Age (years)^†^	56 (45–67)	62 (54–69)	38 (36.5–52.5)	0.004
<52	10 (32.3)	4 (40)	6 (60)	
52–62	10 (32.3)	8 (80)	2 (20)	
>62	11 (35.4)	11 (100)	0	
Debut of diabetes				0.043
Yes	13 (41.9)	7 (53.8)	6 (46.2)	
No	18 (58.1)	16 (88.9)	2 (11.1)	
Previous antihyperglycemic treatment (*n* = 18)				0.79
Oral treatment	7 (38.9)	6 (85.7)	1 (14.3)	
Insulin	8 (44.4)	7 (87.5)	1 (12.5)	
No treatment	3 (11.7)	3 (100)	0	
Previous comorbidities				
Arterial hypertension	12 (38.7)	10 (83.3)	2 (16.7)	0.43
Chronic kidney disease	4 (12.9)	3 (75)	1 (25)	0.73
Obesity	5 (16)	3 (60)	2 (40)	0.58
Cerebrovascular disease	3 (9.7)	3 (100)	0 (0)	0.55
Diabetic ketoacidosis subtype				0.62
Diabetic ketoacidosis	25 (80.7)	20 (80)	5 (20)	
Mixed hyperglycemic state (DKA/HHS)	6 (19.3)	3 (50)	3 (50)	
SaO_2_ on admission^†^	96 (88.9–97.9)	96 (91.1–97.9)	96 (87–98)	0.83
Pa/FiO_2_ on admission^†^	145 (74.9–374)	139 (74.7–252)	225 (88.7–431)	0.32
Laboratory test on admission				
Leukocyte count (×10^3^ cells/μL)^†^	14.3 (11.3–18.7)	13.3 (10.7–18.3)	16.01 (14.0–20.5)	0.24
Neutrophil (%)^†^	87.8 (83–92)	87.8 (81–92.5)	87.4 (84.2–90.5)	0.8
Lymphocyte count (×cells/μL)^†^	717 (549.3–1,144)	702 (417.5–1,011)	1,104 (496–1,207.8)	0.2
Platelet count (×10^3^ cells/μL)^†^	262 (190–375)	260 (222–330)	280 (186–427)	0.55
Hemoglobin (g/L)^†^	13.9 (11.9–14.7)	113.9 (11.8–14.7)	13.7 (12.9–15.1)	0.71
C‐reactive protein (mg/dL)^†^	16.8 (6.1–28.8)	18.59 (8.2–28.9)	7.4 (3.1–25.8)	0.26
Creatinine (mg/dL)^†^	1.33 (0.96–2.72)	1.16 (0.85–2.34)	1.6 (1.1–4.11)	0.31
eGFR (mL/min/1.73 m^2^)	52.6 (25.2–78.5)	65.6 (31–78.6)	45.7 (17.3–61.1)	0.32
Urea (mg/dL)^†^	79 (54–131)	79 (53–131)	79.5 (64–144)	0.62
Ferritin (ng/mL)^†^	1,841 (1,246–2,000)	1,890 (1,301–2,000)	1,466 (796–2,000)	0.39
Lactic dehydrogenase (mg/dL)^†^	711 (569–915)	736 (608–947)	645 (496–779)	0.27
D‐dimer (μg/mL)^†^	2.09 (0.6–2.8)	1.8 (0.6–4.3)	2 (0.57–2.13)	0.45
Arterial blood gas parameters on admission				
Glucose^†^	520 (421–702)	526 (435–667)	442 (345–833)	0.71
pH^†^	7.16 (7.1–7.27)	7.18 (7.1–7.28)	7.14 (7.07–7.21)	0.37
HCO_3_ ^†^	8.6 (6–13.9)	9.1 (6.7–14)	5 (3−8.3)	0.01
Anion gap^†^	17.6 (13.8–19)	16.4 (13.2–18.9)	18.2 (16.8–19.8)	0.18
Osmolarity^†^	302 (290.4–314.7)	300.8 (288.5–314.7)	303.5 (296–333)	0.49
Uncorrected sodium^†^	137 (133–143)	134 (133–141)	142 (131–152)	0.25
Potassium^†^	4.3 (3.1–4.9)	4.6 (3.7– 5)	3.5 (2.9–4.5)	0.26
Chlorine^†^	112 (106–122)	109 (105–115)	121 (107.5–125)	0.2
Lactate^†^	1.5 (1.2–2.5)	1.6 (1.2–3)	1.3 (0.0–1.9)	0.16
Treatment regimen for hyperglycemia				0.29
Continuous insulin infusion	25 (80.7)	17 (68)	8 (32)	
Subcutaneous insulin	6 (19.3)	6 (100)	0	
ICU admission	9 (29)	5 (55.6)	4 (44.4)	0.14

DKA, diabetic ketoacidosis; eGFR, estimated glomerular filtration rate; HHS, hyperglycemic hyperosmolar state; ICU, intensive care unit; Pa/FiO_2_, oxygen partial pressure in arterial blood/fraction of inspired oxygen; SaO_2,_ oxygen saturation.

*P‐values were calculated by Fisher's exact test or Mann–Whitney *U*‐test, as appropriate.

^†^ Median (interquartile range).

Approximately seven out of 10 patients presenting with DKA and COVID‐19 died (74.2% mortality). As expected, mortality was highest among older patients, as recently reported^1^, ^2^. In the USA in a large cohort of patients admitted for DKA, the mortality associated with COVID‐19 was higher compared with those without COVID‐19 (30% vs 5%) in the same study period^1^, another study found a mortality rate of 50%^2^, but in the UK^3^, the reported mortality rate was 5.7%.

We found a large proportion of new diabetes (41.9%), which is higher than other populations^2^, ^3^. This might be related to a high prevalence of underdiagnosed prediabetes (22.4%) and diabetes mellitus (2.8%) in Peru^4^.

Glycemia on admission and laboratory markers of severe COVID‐19 (ferritin, C‐ reactive protein, leukocyte count, etc.) were high (Table 1), which could explain the high mortality. However, in Peru, it could also be due to the negative impact of the gaps in our health system.

In conclusion, among hospitalized patients with DKA and COVID‐19, four of 10 patients showed new diabetes, and levels of glycemia and laboratory markers of severe COVID‐19 were high. Mortality in DKA patients was significantly higher compared with other series.

## References

[1] Pasquel FJ , Messler J , Booth R , *et al*. Characteristics of and mortality associated with diabetic ketoacidosis among US patients hospitalized with or without COVID‐19. JAMA Netw Open 2021; 4: e211091. 33688962 10.1001/jamanetworkopen.2021.1091 PMC7948057

[2] Chamorro‐Pareja N , Parthasarathy S , Annam J , *et al*. Letter to the editor: unexpected high mortality in COVID‐19 and diabetic ketoacidosis. Metabolism 2020; 110: 154301. 32589899 10.1016/j.metabol.2020.154301 PMC7311346

[3] Armeni E , Aziz U , Qamar S , *et al*. Protracted ketonaemia in hyperglycaemic emergencies in COVID‐19: a retrospective case series. Lancet Diabetes Endocrinol 2020; 8: 660–663. 32621809 10.1016/S2213-8587(20)30221-7 PMC7329282

[4] Seclen SN , Rosas ME , Arias AJ , *et al*. Prevalence of diabetes and impaired fasting glucose in Peru: report from PERUDIAB, a national urban population‐based longitudinal study. BMJ Open Diabetes Res Care 2015; 3: e000110. 10.1136/bmjdrc-2015-000110 PMC4620143 26512325

